# Synergistic Effects of Diatoms on Intumescent Flame Retardant High Impact Polystyrene System

**DOI:** 10.3390/polym14204453

**Published:** 2022-10-21

**Authors:** Fuhua Lin, Mi Zhang, Xiangyang Li, Shuangdan Mao, Yinghui Wei

**Affiliations:** 1 School of Materials Science and Engineering, Taiyuan University of Science and Technology, Taiyuan 030024, China; 2 Shanxi Province Institute of Chemical Industry Co., Ltd., Jinzhong 030621, China; 3 Key Laboratory of Renewable Energy, Chinese Academy of Sciences, Guangzhou 510640, China; 4 Shanxi Advance Technology Low Carbon Industry Research Institute Co., Ltd., Taiyuan 030021, China

**Keywords:** high impact polystyrene (HIPS), flame retardancy, mechanical property, intumescent flame retardant (IFR), char

## Abstract

In this work, aiming to improve the flame retardancy performance of high impact polystyrene (HIPS), HIPS compounds were synthesized with the addition of intumescent flame retardant (IFR: mass ratio of APP and PER was 3:1) and diatoms into HIPS matrix by melt blending method. It was found the IFR/diatoms system exhibited high flame retardant efficiency and catalytic carbonization effect to HIPS matrix in the burning process. The LOI value of HIPS-2 compound with the addition of 28 wt% IFR and 2 wt% diatoms was increased to 29.0% and passed V-0 rating. The value of PHRR for HIPS-2 compound is about 460.58 kW/m^2^ compared with 937.22 kW/m^2^ of pure HIPS and the value of THR for HIPS-2 compound is about 32.9 MJ/m^2^ compared with 62.7 MJ/m^2^ of pure HIPS, suggesting that the addition of IFR/diatoms system can decrease the values of PHRR and THR, which shows the synergistic effect between IFR and diatoms on reducing heat release. The 21.9% reduction in Av-EHC and 41.4% reduction in TSP seen on introducing an IFR/diatoms system indicates effective smoke suppression, which potentially would significantly reduce the death rate in real fire accidents. The TG-IR results indicated that the IFR/diatoms flame retardant system functioned in the gas phase to suppress the flame. The SEM images showed the char residue produced was more compact and continuous, which suggests that the IFR/diatoms flame retardant system exhibits barrier and catalytic effects to block heat transferring and promote char forming. The tensile strength and impact strength of HIPS-2 compound were 22.95 MPa and 2.63 KJ/m^2^, respectively. The tensile strength and impact strength were increased by 34.13% and 19.55% compared with that of pure HIPS.

## 1. Introduction

High-impact polystyrene (HIPS) is one of the most widely used thermoplastic materials with good dimensional stability, excellent electrical insulation, chemical corrosion resistance and convenience of processing and molding [1,2,3]. Due to these characteristics, it is employed in the fields of housing packaging of household appliances, electronic products, military materials and other industries [4,5]. The main application field of HIPS in the home is the electrical appliance industry, which accounts for about 60% of the total consumption. Despite its many advantages, HIPS belongs to flammable materials, which have a low oxygen index (only 18.0%) and a serious dripping issue during burning, resulting in severe threat to life and property safety [6]. Therefore, it is particularly critical to endow HIPS with good flame retardant properties.

Intumescent flame retardant (IFR) is a kind of halogen-free flame retardant containing P, C and N elements, which are generally composed of three parts: acid source (dehydrating agent), carbon source (char-forming agent) and gas source (foaming agent) [7,8,9]. At the present stage, on account of the low smoke emission, low toxicity and high flame retardant efficiency, IFR has attracted extensive attention from researchers [10]. The most common IFR system is mainly composed of ammonium polyphosphate (APP), pentaerythritol (PER), where PER serves as carbon source and APP plays a role in acid source and gas source [11,12,13]. Li et al. [14] used APP/PER as an effective additive to promote the flame retardancy of polypropylene (PP). The incorporation of APP/PER resulted in improving the catalytic charring ability and smoke suppression during burning. The results indicated that the LOI value reached the maximum value at around 27.8% and the UL-94 test reached V-0 rating, when the ratio of APP/PER was 1.5:1 (wt/wt). However, the bad compatibility between the intumescent flame retardants and polymeric matrix will result in the migration in the surface of polymers, and a decrease in mechanical properties. In response to the above disadvantages, lots of studies focused on the use of various synergistic agents, such as organ-montmorillonite [15], CaCO_3_ [16], organoboron siloxane [17], and metal compounds [18]. It has been found that the Si-containing compounds can be used as synergistic flame retardants for polymers by providing additional effects such as the formation of an expanded structure and the increase in fire-proofing and the barrier effect. Nour F. Attia et al. [1] added rice husk ash into the polystyrene and diammonium hydrogen orthophosphate composite; the results indicated the synergism effect in terms of flame retardant property. Nour F. Attia et al. [2] prepared the polypyrrole nanoparticles with an average nanoparticle size of 161 nm, where the PHRR and THR of the polymer nanocomposites were reduced, achieving 78 and 31% reduction, respectively. Diatomite, as a king of fossil-like non-metallic minerals, mainly contains SiO_2_ with little Fe_2_O_3_, Al_2_O_3_, MgO, etc. Diatomite is widely distributed in the world and has the characteristics of green materials, large porosity, high permeability, and stable chemical properties. In the flame retardant field, diatomite is often used as a synergistic flame retardant in combination with other flame retardants. Cavodeau et al. [19] studied the effects of diatomite as a synergistic agent on the flame retardancy in aluminum hydroxide/ethylene-vinyl acetate composites (ATH/EVA). The cone calorimeter tests indicated the presence of diatomite could promote to form the homogenous and compact char layer, thus resulting in the decrease in heat release rate, total heat release, carbon monoxide and carbon dioxide. Some research has shown that the addition of a small amount of diatomite as a synergistic flame retardant has a remarkable trend of enhancing the flame retardant performance of polymers [20].

In this paper, the intumescent flame retardant (IFR) and Si-containing flame retardant diatoms were introduced to an HIPS matrix to improve the flame retardant property by melt blending method. The dispersion of the flame retardant system in an HIPS matrix was characterized by scanning electron microscopy (SEM). The mechanical property and flame retardancy of the HIPS compounds were investigated by tensile strength tests, LOI, vertical burning test (UL-94) and TG-FTIR, respectively. In addition, the flame-retardant mechanism of the HIPS compounds was studied by SEM, Raman spectroscopy, XRD and Fourier transform infrared spectroscopy (FTIR) of the char residues.

## 2. Materials and Methods

### 2.1. Materials

High impact polystyrene (HIPS, PH-888G) was provided from Qimei Chemical Co., Ltd., (Zhenjiang, China). Diatoms were obtained from Jiuying New Material Co., Ltd., (Shanghai, China). Ammonium polyphosphate (APP) was purchased by Wenjiang Chemical Reagent Co., Ltd., (Guangdong, China). Pentaerythritol (PER) was received from Lingfeng Chemical Reagent Co., Ltd., (Shanghai, China).

### 2.2. Treatment of Diatoms

**Figure 1:**
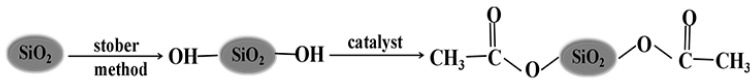
The treatment of diatoms.

Diatomite was crushed by a rotary mill to obtain a powder with a measured D_50_ of about 5 mm. Alkaline silica sol (SiO_2_) was prepared according to the Stöber method. The prepared alkaline silica sol was mixed with acetic acid (CH_3_COOH) in flask by using stannous octoate as a catalyst, which was reacted at 100 °C for 1 h. The above reactants were centrifuged with chloroform and dried. The processing of treatment of diatoms is shown in Figure 1.

### 2.3. Preparation of IFR/Diatoms/HIPS Flame Retardant Compounds

Firstly, IFR flame retardant was prepared by the mixture of APP and PER (mass ratio was 3:1) and dried in an oven at 60 °C for 24 h. Then, HIPS, IFR and diatoms were added into a micro two-screw extruder (SJSZ-10, Ruiming Plastics Machinery Co., Ltd., Wuhan, China) and extruded twice to ensure they were evenly mixed. The temperature of four heating sections of the extruder was 200, 210, 220, 200 °C, respectively. The obtained mixture was injection molded into appropriate specimens for tests by a SZ-15 micro injection molding machine (Ruiming Plastics Machinery Co., Ltd., Wuhan, China). The injection temperature was 230 °C and the injection pressure was 0.5 MPa. The formula of the IFR/diatoms/HIPS flame retardant compounds is listed in Table 1.

### 2.4. Measurement and Characterization

The fracture surface morphologies of the HIPS compounds were analyzed using scanning electron microscopy (SEM, S4800, Tokyo, Japan).

The mechanical property of the HIPS compounds was measured with the molded dumbbell-shaped test specimens. The samples with dimensions of 10 mm × 3 mm × 3 mm was in accordance with ASTM D638 on a CMT tensile tester (Sans, Shenzhen, China). The measurements were performed with a rate of 10 mm/min at room temperature. Each spline was measured at least 5 times.

Limiting oxygen index (LOI) tests were conducted on a JF-3 oxygen index meter (Jiangning Analysis Instrument Co., Nanjing, China) with the sample dimension of 80 mm × 10 mm × 4 mm according to GB/T10707-1989. UL-94 burning test was performed on the samples of 80 mm × 10 mm × 4 mm in size with CFZ-3 instrument (Jiangning Analysis Instrument Co., Nanjing, China).

Thermogravimetric analysis (TGA, DTG-60 thermoanalyzer, Shimadzu, Japan) was carried out from ambient temperature to 600 °C in nitrogen flow (20 mL/min) at a heating rate of 20 °C/min. The samples used in this measurement were kept within 3–5 mg in ceramic crucibles.

Thermogravimetric analysis coupled to Fourier transform infrared spectroscopy (TG-IR) measurements were performed from 40 to 600 °C in N_2_ atmosphere (20 °C/min, flow rate of 50 mL/min) with a STA 409PC instrument (Netzsch, Bayern, Germany), combined with a Thermo Nicolet iS10 FTIR spectroscope (Thermo Scientific Inc., Norwood, MA, USA). The resolution of IR spectra was 4 cm^−1^ and the range was 4000–400 cm^−1^.

Cone calorimeter (Fire Testing Technology, East Grinstead, UK) tests were performed based on GB/T 16172-2007 standard under an external heat flux of 35 kW/m^2^. The sample sizes for the cone calorimeter test were 100 mm × 100 mm × 3 mm.

The carbonaceous char morphologies of the HIPS compounds were analyzed using scanning electron microscopy (SEM, JEOL-6380LV, Tokyo, Japan).

Raman spectra was tested on a Renishaw Invia laser Raman spectrometer (England) at the range from 100 to 3500 cm^−1^ by He laser beam excitation to analyze the graphitization degree of char residues.

X-ray diffraction (XRD, Bruker D8 Advance diffractometer, Germany) measurements were performed with Cu Kα radiation (0.15418 nm) to characterize char residues. The condition was at 40 kV and 40 mA. The scanning rate is 0.1 °/s and the scanning range was 5–80°.

Fourier transform infrared spectra (FTIR, Shimadzu FTIR-8400S spectrometer, Japan) was recorded at a range of 4000–400 cm^−1^ with a resolution of 4 cm^−1^ to observe the structure of char residues. KBr powder and sample were mixed at a ratio of 1:100 to grind and tablet.

## 3. Result and Discussion

### 3.1. SEM Photographs of the HIPS Compounds

**Figure 2:**
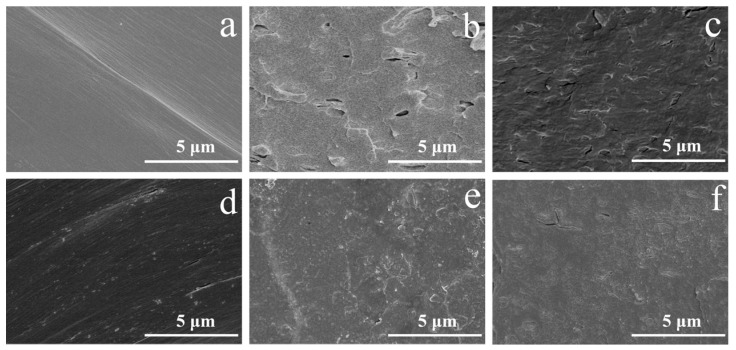
The SEM photographs of the impact fracture surface for the HIPS compounds: (**a**) HIPS; (**b**) HIPS/IFR; (**c**) HIPS-1; (**d**) HIPS-2; (**e**) HIPS-3; (**f**) HIPS-4.

**Figure 3:**
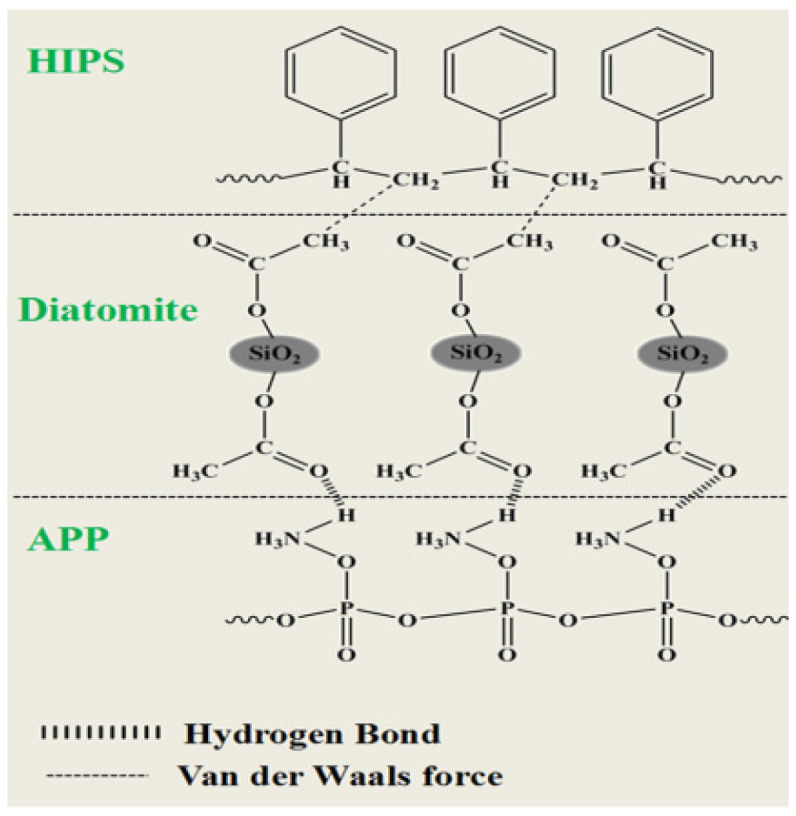
The diagram of IFR/diatoms system and HIPS matrix.

The SEM photographs of the HIPS compounds impact fracture surface are shown in Figure 2. The fractured surface of neat HIPS is quite smooth, with no-crack morphology. The HIPS matrix phase and IFR flame retardant phase can be clearly seen in Figure 2b. There are a large amount of holes and cracked particles on the surface, suggesting poor dispersion and weak interfacial interactions between HIPS and IFR flame retardant. As can be observed in Figure 2c, the number of holes and cracked particles is decreased obviously in the impact fracture surface after adding 1 wt% diatoms. Increasing the diatoms loading to 2 wt% (Figure 2d), the IFR and diatoms flame retardant is uniformly dispersed in HIPS matrix and a flat fracture morphology without obvious defects is formed, which is due to the interpretation that the IFR/diatoms flame retardant system greatly reduces the interfacial tension and causes the enhanced interfacial adhesion. Since diatoms contain high molecular weight polydimethylsiloxane, there is van der Waals force between its pendant CH_3_ and HIPS matrix, and hydrogen bonding between NH^4+^ on the surface of APP and the O atom of the polydimethylsiloxane main chain (Figure 3), thereby improving the interface compatibility between HIPS and IFR [21,22]. However, the HIPS-3 and HIPS-4 compounds exhibit a rough morphology with some defects (Figure 2e,f). This may be due to the excessive loading of diatoms.

### 3.2. Mechanical Property of the HIPS Compounds

**Figure 4:**
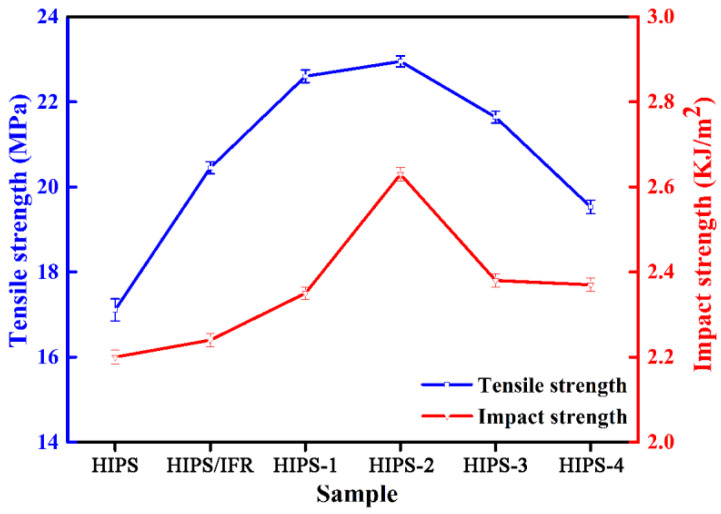
The tensile strength and impact strength of the HIPS compounds.

The tensile strength and impact strength of the HIPS compounds are shown in Figure 4. It can be seen that the tensile strength and impact strength of pure HIPS are 17.11 MPa and 2.20 KJ/m^2^, respectively. After 30 wt% IFR adding, the tensile strength and impact strength of the HIPS/IFR compound increase to 20.45 MPa and 2.24 KJ/m^2^, increasing by 19.52% and 1.82%, respectively. The reason for the phenomenon is that IFR can be used as a stress concentration point in the HIPS matrix. When the HIPS compound is subjected to external force, the IFR particles can cause a large number of crazes and shear bands, which can absorb more energy and improve mechanical property [23,24]. In addition, when diatoms are added, the tensile strength and impact strength of the HIPS compounds increase first, and then decrease. When the addition of diatoms is 2 wt%, the tensile strength and impact strength reach the maximum value of 22.95 MPa and 2.63 KJ/m^2^. The tensile strength and impact strength are increased by 34.13% and 19.55% compared with that of pure HIPS. These dates are increased by 12.22% and 17.41% compared with that of HIPS/IFR compound. This phenomenon proves that the diatoms can evenly disperse in the HIPS matrix and improve the inter-molecular interaction force when a proper amount of diatoms is added. As a result of the surface energy of diatoms being high, the serious agglomeration of diatoms in the HIPS matrix happens when the amount of diatoms is excessive [25]. This phenomenon hinders the stress transfer and reduces the mechanical property of the HIPS compounds.

### 3.3. Flame Retardancy of the HIPS Compounds

**Figure 5:**
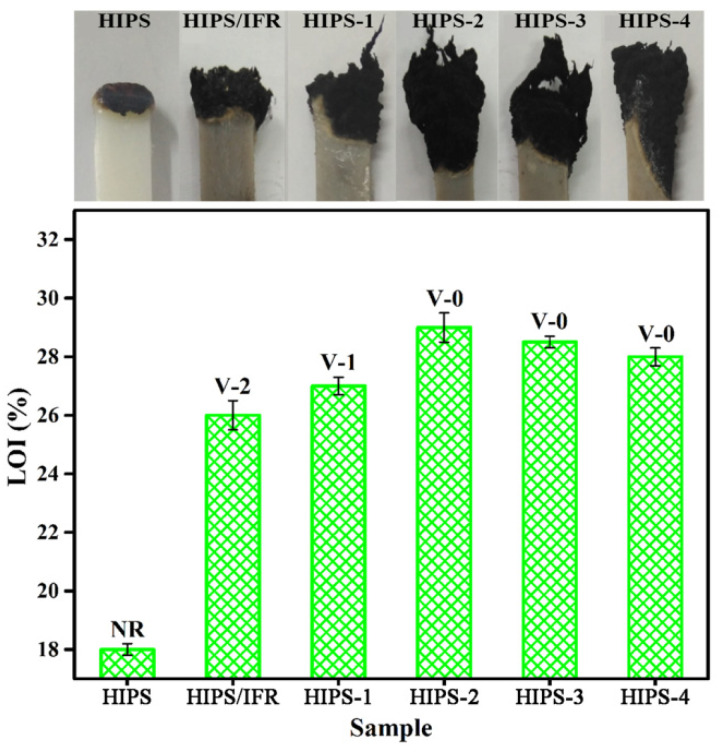
The digital photos after LOI tests, the LOI and UL-94 results of the HIPS compounds.

The LOI and UL-94 vertical burning test are diffusely applied to assess the flame retardant property of polymers and the test results are shown in Figure 5 [26]. Pure HIPS has an intrinsic inflammability with a LOI value of about 18.0% and no rating of UL-94. Moreover, Figure 5 shows the digital photos of the HIPS compounds after LOI tests. As can be observed, the pure HIPS burns with serious droplet phenomenon and generates little carbon residue, which greatly limits its practical application in some fields. Compared with pure HIPS, the LOI values of HIPS/IFR compounds with the loading of IFR are significantly improved. It can be seen that the LOI value of HIPS/IFR compounds is increased rapidly to 26.0% when adding 30 wt% IFR, which can be attributed to the flame retardant effect of IFR. In addition, the droplet phenomenon is significantly improved and the char residue yield is relatively increased. The addition of diatoms leads to further improvement in flame retardancy. The LOI value of HIPS-2 compound with the addition of 28 wt% IFR and 2 wt% diatoms is increased to the highest value, of 29.0%, and passes V-0 rating. A large amount of char residue is formed after burning, which indicates that diatoms and IFR have a higher synergistic effect on flame retardancy of HIPS.

### 3.4. TGA and TG-IR of the HIPS Compounds

**Figure 6:**
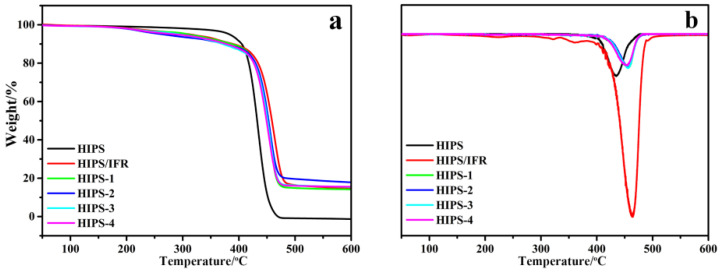
The TGA (**a**) and DTG (**b**) results of the HIPS compounds.

TG is usually used to study the thermal degradation behavior during the thermal degradation process. The effect of IFR and diatoms on the thermal stability for HIPS is characterized by TGA under nitrogen atmosphere and the corresponding results are shown in Figure 6 and Table 2. Table 3 exhibits the related parameters, including T_5wt%__,_ T_max_ and char residue yield at 600 °C. It can be observed that pure HIPS and HIPS compounds undergo a one-step thermal decomposition stage, which occurs at 400–500 °C. The onset degradation temperature (T_5wt%_) is defined as the temperature where 5% weight loss occurs. With the addition of IFR and diatoms, HIPS/IFR compounds show T_5wt%_ inferior to that of pure HIPS, which is initiated by the degradation in IFR and diatoms, where the produced phosphoric acid and SiO_2_ solid acid have a strong catalytic carbonization effect on HIPS compounds, which in turn accelerates the degradation rate [3]. Theoretically, almost all the chains of HIPS are cracked into combustible gas during burning, and there is no residual carbon. It is apparent that a higher residual weight is produced during burning after introducing IFR and diatoms into the HIPS matrix, which indicates that the thermal decomposition products of IFR and diatoms may promote the carbonization of the HIPS matrix, thereby accelerating the formation of carbonaceous char.

**Figure 7:**
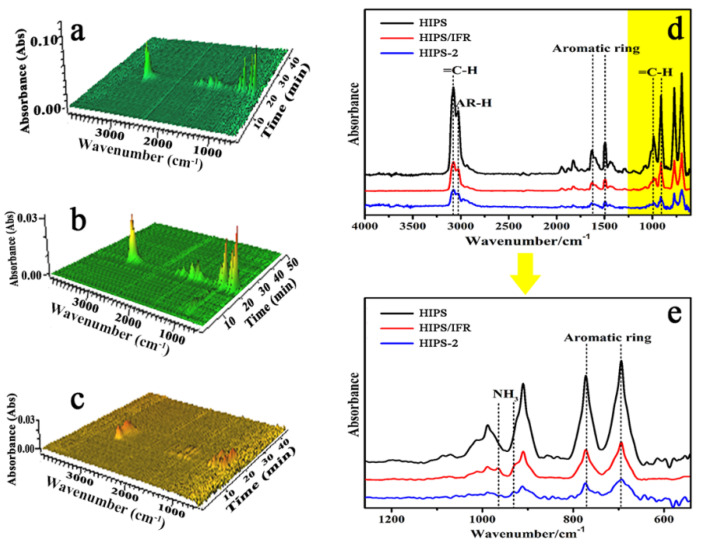
The TG-TR results of the HIPS compounds: (**a**) 3D TG-IR spectra of pure HIPS; (**b**) 3D TG-IR spectra of pure HIPS/IFR; (**c**) 3D TG-IR spectra of HIPS-2; (**d**,**e**) FTIR spectra of pyrolysis gases for pure HIPS, HIPS/IFR and HIPS-2 at the maximum decomposition rate.

In order to further analyze the gas flame retardant mechanism of the IFR/diatoms system, the gaseous pyrolysis products released during the thermal degradation in the HIPS compounds are monitored by TG-IR, which is of great significance for the further understanding of thermal degradation behavior. Figure 7a–c presents the 3D images of gaseous products released by pure HIPS and HIPS compounds during the heating process, respectively. As depicted in Figure 7a–c, compared with pure HIPS, the total gas emission of HIPS/IFR and HIPS-2 compounds is significantly reduced, which further proves that the combination of IFR and diatoms can obtain a synergistic effect on the suppression of gaseous products generated from the burning process.

Figure 7d,e shows the FTIR spectra of gas products for pure HIPS, HIPS/IFR with only IFR and HIPS-2 with IFR and diatoms. Obviously, there is a weakened intensity for absorption peaks of HIPS/IFR and HIPS-2 compound compared to that of pure HIPS. The stretching vibration of =C-H is detected at 3077 cm^−1^ and the bending vibration is located at 983 and 908 cm^−1^, respectively [27]. The peaks at around 1637, 1495, 766 and 691 cm^−1^ are attributed to the characteristic vibration of an aromatic ring, indicating that the main decomposed gases of HIPS are monomer, dimer and trimer of phenyl alkenyl [28]. In addition, the vibrating absorption peaks appearing at around 965 and 931 cm^−1^ in curves are related to the vibration mode of NH_3_, suggesting that NH_3_ is released to the gas phase by the decomposition of APP after burning.

**Figure 8:**
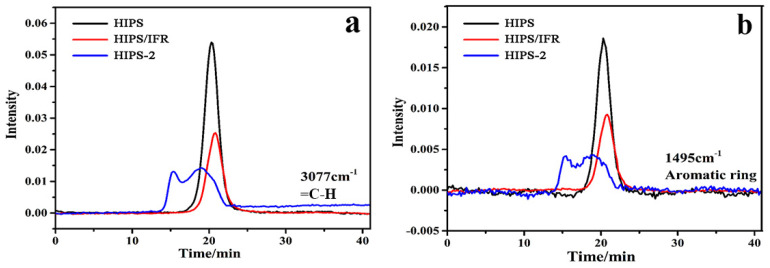
The absorbance of volatile products of the HIPS compounds: (**a**) =C-H; (**b**) aromatic ring.

To compare the peak quantitatively, the absorbance intensity of =C-H peak (3077 cm^−1^) and the aromatic ring peak (1495 cm^−1^) are shown separately in Figure 8a,b. As depicted in Figure 8a, the appearance of double peaks for HIPS-2 is ascribed to the fact that the incorporation of diatoms can cause the early decomposition of aromatic compounds and unsaturated hydrocarbons during the pyrolysis process, which also has a catalytic effect on HIPS matrix and can quickly form a continuous and dense barrier layer on the surface of HIPS compounds, thereby suppressing the thermal decomposition of internal material further. Moreover, the absorption intensity of HIPS/IFR and HIPS-2 compounds is significantly reduced compared with that of pure HIPS, suggesting that the addition of IFR and diatoms can effectively reduce the amount of pyrolysis products in gas phase. Similar results are observed for the aromatic ring, as shown in Figure 8b.

### 3.5. CCT of the HIPS Compounds

**Figure 9:**
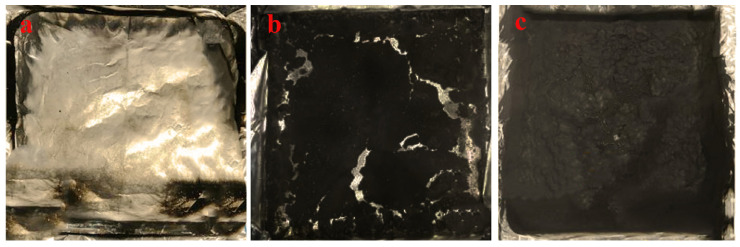
Digital photos of the char residues after the CCT test: (**a**) HIPS; (**b**) HIPS/IFR; and (**c**) HIPS-2.

Cone calorimetry is a high-efficiency technique to evaluate the fire behaviors of polymers, which gives out the parameters such as the peak heat release rate (PHRR), the total heat release (THR), the time to ignition (TTI), the fire growth rate (FIGRA), the average effective heat of combustion of volatiles (Av-EHC) and the total smoke production (TSP), as shown in Table 3. Figure 9 displays digital photos of the char residues for the HIPS compounds after the CCT test. There is a little amount of cracked char residue for pure HIPS (Figure 9a), where a large amount of char residues are generated and an extremely compact char layer is achieved for the HIPS-2 compounds after burning (Figure 9c). Peak heat release rate (PHRR) and total heat release (THR) play a crucial role in investigating the heat release process and fire safety of samples. Pure HIPS has an intrinsic inflammability with PHRR value of 937.22 kW/m^2^ and THR value of 62.7 MJ/m^2^, respectively. It is evident that the inclusion of IFR/diatoms system can decrease the values of PHRR and THR higher than that of single IFR, indicating the synergistic effect between IFR and diatoms on reducing heat release. The value of TTI for pure HIPS is about 40 s, which is due to the heat generated after burning being more likely to diffuse into the polymers, so that the time required for the surface layer to achieve thermal degradation and release flammable volatiles becomes longer. After adding the flame retardant, the time to ignition (TTI) of samples is decreased to a certain extent. This could be mainly due to the early decomposition of IFR flame retardant additives [29]. Effective heat of combustion (EHC), which is defined as the ratio of HRR to mass loss rate measured at a certain point, implies the burning degree of volatile gases is generated from materials. Incorporating IFR flame retardant into an HIPS matrix leads to a slight improvement in flame retardancy in terms of Av-EHC and TSP due to its poor dispersion level and weak interfacial interactions with the HIPS matrix. The 21.9% reduction in Av-EHC, and the 41.4% reduction in TSP seen on introducing the IFR/diatoms system, indicates effective smoke suppression, which potentially would significantly reduce the death rate in real fire accidents. Furthermore, FIGRA is usually applied to assess the fire risks of polymers, where a lower value suggests a higher fire safety. After adding the IFR/diatoms system, the HIPS-2 compound has an evident reduction in FIGRA value. The CCT results indicated the IFR/diatoms system is expected to be a useful flame retardant for the HIPS matrix.

### 3.6. The SEM, Raman, XRD and FTIR Curves of Carbonaceous Char for the HIPS Compounds

**Figure 10:**
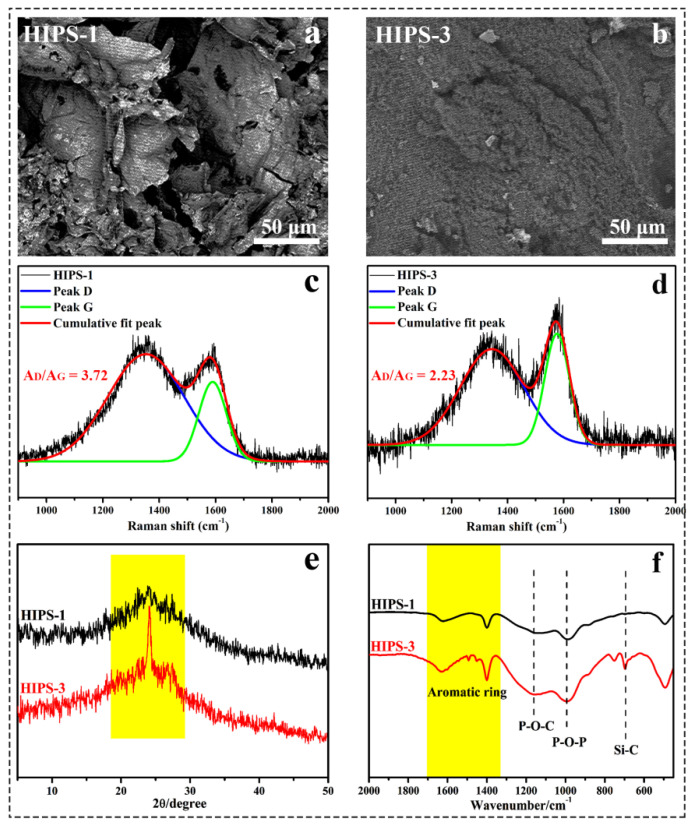
The SEM images (**a**,**b**), Raman curves (**c**,**d**), XRD (**e**) and FTIR (**f**) curves of carbonaceous char for the HIPS-1 and HIPS-3 compounds.

The carbonaceous char produced during the thermal degradation of the HIPS compounds are monitored by SEM images, Raman, FTIR and XRD, which is of great significance for further understanding the condensed flame retardant mechanism of the IFR/diatoms system. Figure 10a,b show the morphology of carbonaceous char for the HIPS/IFR and HIPS-2 compound, respectively. With the incorporation of IFR, it shows a loose and porous structure with lots of holes and cracks, which act as channels for the transmission of heat and oxygen. While adding the IFR and diatoms, a continuous and dense carbonaceous char with almost no obvious holes is observed, which is sufficient to protect the polymer layers inside the compound. Figure 10c,d present the Raman curves fitted by Gaussian method for the HIPS compounds after burning [30]. The peak located at 1340 cm^−1^ (peak D) is identified as an amorphous characteristic peak of graphite and the peak centered at 1578 cm^−1^ (peak G) is identified as an ordered characteristic peak of graphite [31]. The ratio (AD/AG) is used to estimate the graphitization degree of carbonaceous char. More importantly, the ratio is inversely proportional to an in-plane microcrystalline size, where a smaller size of carbonaceous microstructures indicates a better shield effect of carbonaceous char from burning and better flame retardancy. Notably, the value of HIPS-2 compound is decreased to 2.23 compared to 3.72 of HIPS/IFR as the loading of diatoms, suggesting that the addition of diatoms is beneficial to promote the formation of a smaller size of carbonaceous structure and highly ordered char layer. Considering the XRD pattern of the HIPS-2 compound (Figure 10e), there is a strong and broad peak around 2θ = 24.3°, which corresponds to the (002) diffraction peak of graphite crystals [32]. Besides, the diffraction peak at 2θ = 26.6° is attributed to the (011) characteristic peak of quartz phase [33]. The XRD results imply that the presence of diatoms makes a change to the structure of the carbonaceous char in the burning process, which can effectively enhance the stability of the carbonaceous char and improve the flame retardant efficiency of HIPS. FTIR analysis is further performed to provide detailed information on the carbonaceous char. In the case of FTIR spectra for HIPS compounds (Figure 10f), the absorption peaks within the range of 1630 to 1400 cm^−1^ correspond to the skeleton characteristic peak of the aromatic ring [34]. The peaks appearing at about 1161 and 996 cm^−1^ can be assigned to the characteristic vibrations of P-O-C and P-O-P in the phosphates compounds. Moreover, the absorption peak near 694 cm^−1^ is attributed to the characteristic vibration of Si-C group. The FTIR results suggest that the carbonaceous char of HIPS-2 compound mainly consist of complexes with silicon-containing compounds, phosphates and aromatic ring compounds, which can effectively have a shielding effect and prevent the inner compound from further burning, leading to a high flame retardant efficiency.

### 3.7. Analysis of Flame Retardant Mechanism for the HIPS Compounds

**Figure 11:**
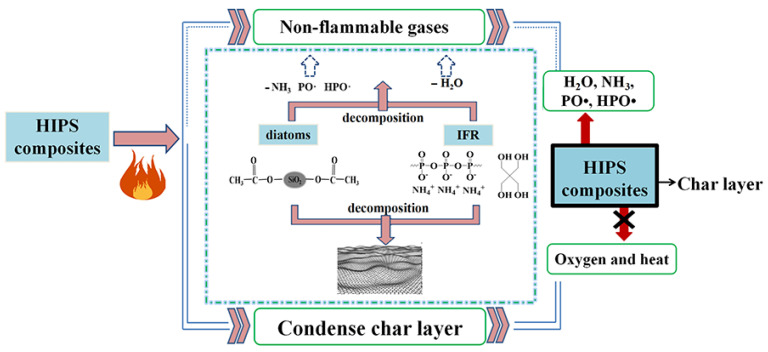
Possible flame retardant mechanism of the HIPS compounds.

A continuous and compact carbonaceous char is the key to decreasing the contact between oxygen and heat, which can effectively protect the inner polymer without decomposition. According to the above analysis of carbonaceous char, the general flame retardant mechanism is exhibited in Figure 11. The abnormally excellent flame retardant performance for the HIPS compounds is the result of the synergistic effect between IFR and diatoms in the gas phase and the condensed phase, respectively. In this system, the chemical reactions are complex. During burning, in the gas phase, APP, acting as the gas source, first decomposes and generates a large amount of non-flammable gases such as H_2_O and NH_3_, which can reduce the concentration of combustible gases around the surface of HIPS compounds. Simultaneously, the generated free radicals (such as PO· and HPO·) can capture the active free radicals (such as H and OH·) in the pyrolysis process, which effectively inhibits the burning chain reaction, thereby suppressing the development of burning and achieving the purpose of preventing burning. Moreover, during heating, the main gaseous product makes the mixture of the carbonaceous residue swell, leading to the formation of an intumescent char layer. In the condensed phase, with the rapid spreading of the surface temperature, the IFR/diatoms flame retardant system can migrate to the surface of HIPS compounds and degrade to form a protective barrier with high thermal stability. On the one hand, the decomposition of APP and diatoms to produce phosphoric acid and SiO_2_ solid acid has a strong catalytic carbonization effect on HIPS compounds, which accelerates the oxidative dehydrogenation cross-linking-charring process and promotes the formation of carbonaceous char layer on the surface of HIPS compounds [35]. On the other hand, theoretically, with the loading of diatoms, the SiO_2_ produced by the thermal decomposition of diatoms can react with the decomposition products of APP to form a high thermal stability structure with a silicon-phosphate cross-linked substance [36,37], which is able to act effectively as a physical barrier to reduce the mass/heat exchange on the surface, and prevent the escape of volatiles, thus resulting in the significant improvement of flame retardancy for the HIPS compounds [38].

## 4. Conclusions

In this work, the IFR/diatoms system could significantly promote the flame retardant property and mechanical property of HIPS. The results of LOI and TG-IR confirmed that the synergistic effect between IFR and diatoms on the flame-retardant HIPS compounds could effectively reduce the amount of pyrolysis products in the gas phase. Meanwhile, the LOI value and UL-94 rating for the HIPS-2 compound containing 28 wt% IFR and 2 wt% diatoms increased by 30.9% and passed V-0 rating, respectively, when compared to that of pure HIPS. The results of SEM images, Raman, XRD and FTIR curves of carbonaceous char revealed that the HIPS compounds could generate a continuous and compact carbon layer after burning, which hindered the transmission of oxygen and heat, thus effectively protecting the HIPS compounds. In addition, the HIPS-2 compounds had a higher tensile strength (22.95 MPa) and impact strength (2.63 KJ/m^2^) than that of pure HIPS.

## Figures and Tables

**Table 1:** The formula of the IFR/diatoms/HIPS flame retardant compounds.

Samples	HIPS	HIPS/IFR	HIPS-1	HIPS-2	HIPS-3	HIPS-4
HIPS/wt%	100	70	70	70	70	70
IFR/wt%	0	30	29	28	27	26
diatoms/wt%	0	0	1	2	3	4
HIPS/wt%	100	70	70	70	70	70

**Table 2:** The related parameters of the HIPS compounds.

Samples	HIPS	HIPS/IFR	HIPS-1	HIPS-2	HIPS-3	HIPS-4
T_5wt%_/°C	383.2	292.9	292.5	280.6	284.8	285.1
T_max_/°C	434.9	463.9	455.2	456.7	455.9	453.9
char residue/%	0.0	14.6	14.2	17.8	15.4	15.6

**Table 3:** The cone calorimetry test data of the HIPS compounds.

Samples	HIPS	HIPS/IFR	HIPS-2
PHRR/(kW/m^2^)	937.22	530.71	460.58
THR/(MJ/m^2^)	62.7	48.9	32.9
TTI/s	40	35	23
Av-EHC/(MJ/kg)	32.4	29.6	25.3
TSP/(m^2^)	29	22	17
FIGRA/(kW/m^2^_·_s)	6.8	5.5	4.9

## Data Availability

Not applicable.
